# Challenges in Evaluating a Community-Level Intervention to Address Root Causes of Youth Violence

**DOI:** 10.1007/s11121-024-01678-7

**Published:** 2024-05-11

**Authors:** Krista R. Mehari, Phillip N. Smith, Benterah C. Morton, Joél L. Billingsley, Jasmine N. Coleman, Albert D. Farrell

**Affiliations:** 1 Department of Psychology and Human Development, Vanderbilt University, Nashville, TN USA; 2 Department of Psychology, University of South Alabama, Mobile, AL USA; 3 Department of Psychology, University of Tennessee, Knoxville, TN USA; 4 Clark-Hill Institute for Positive Youth Development & Department of Psychology, Virginia Commonwealth University, Richmond, VA USA

**Keywords:** Youth violence, Suicide, Methodology, Ecological systems, Evaluation

## Abstract

Violence disproportionately impacts Black American youth, representing a major health disparity. Addressing the possible root causes of structural inequities to reduce violence may increase the impact of prevention strategies. However, efforts to evaluate the impact of such interventions pose numerous methodological challenges, particularly around selecting an effective evaluation design to detect change at the community level, with adequate power and sampling, and appropriate constructs and measurement strategies. We propose a multiple baseline experimental design to evaluate the impact of a community-level youth violence and suicidality prevention strategy. A multiple baseline experimental design with multiple community units balances the need for scientific rigor with practical and values-based considerations. It includes randomization and plausible counterfactuals without requiring large samples or placing some communities in the position of not receiving the intervention. Considerations related to the conceptualization of the logic model, mechanisms of change, and health disparity outcomes informed the development of the measurement strategy. The strengths and weaknesses of a multiple baseline experimental design are discussed in comparison to versions of randomized clinical trials. Future health disparity intervention evaluation research will benefit from (1) building a shared sense of urgent public need to promote health; (2) respecting the validity of values- and partnership-based decision-making; and (3) promoting community-based and systems-level partnerships in scientific grant funding. The described study has been registered prospectively at clinicaltrials.gov, Protocol Record 21–454.

**Supplementary Information:**

The online version contains supplementary material available at 10.1007/s11121-024-01678-7.

## Introduction

Violence disproportionately impacts Black American youth. Interpersonal violence has been a long-standing health disparity, accounting for 42% of the deaths of Black American youth between the ages of 15 and 19, compared with 8% of the deaths of White American youth within that age group (CDC, 2020). Although suicide has historically disproportionately impacted White Americans compared to Black Americans, it has shown startling increases among Black American youth. Suicides among Black youth ages 5 to 14 years increased over 250% between 2001 and 2020 (CDC, 2020). This increase was notably higher for Black youth compared to White youth, and the suicide rate was *higher* among Black youth than among White youth in 2020 (CDC, 2020). The long-standing health disparity of interpersonal violence and the disproportionate increase in suicidality among Black American youth point to an urgent need to address root causes of disparities.

Interpersonal violence (or other-directed violence) and suicide (or self-directed violence) have historically been studied independently. However, a social-ecological framework encourages us to consider not just proximal antecedents to intentional injury, but the historical, macrosystemic, and microsystemic factors that may represent shared root causes of violence and suicide (Bronfenbrenner, 1977). Structural inequities may be a shared root cause of violence and suicide, and addressing the structural inequities to which Black youth are disproportionately exposed may reduce both violence and suicide in similar ways. Black youth experience inequities at multiple levels, including structural racism—embedded patterns of racial discrimination in which policies and normative practices maintain or promote disparities—in many systems, including education and law enforcement (Thompson & Neville, 1999). Exposure to racism predicts violence perpetration (Benner et al., 2018) and suicidal ideation among youth (O’Keef et al., 2015; Polanco-Roman et al., 2019). The relation between racism exposure and subsequent interpersonal violence and suicidality is partially explained by general strain theory, which posits that unjust treatment leads to poor mental health, such as anger, traumatic stress, distress, and depression, which lead to violence (e.g., Agnew, 2001). Racism erodes social bonds through systematic injustice, exclusion, and dehumanization. Social erosion, in turn, threatens the belongingness and social support that deter violence (Burt et al., 2012). Therefore, promoting equity across systems may strengthen social bonds and well-being, which may reduce violence.

Efforts to evaluate the impact of systems-level interventions to reduce health disparities pose numerous methodological challenges. We illustrate these challenges and methods for addressing them by discussing the decision-making behind the study design of a grant-funded project. We describe a multiple baseline design to evaluate a multisystemic intervention intended to reduce disparities in interpersonal violence and suicidality. We discuss key issues, such as characteristics of the intervention that shaped the design of the evaluation, systems-level measurement of change, and approaches to analysis. An exemplar design, of course, would include plentiful resources that would allow for a greater length of evaluation and a greater number of communities, but we present a design that is acceptable to communities and feasible based on typical funding constraints. It has strengths and weaknesses that reflect decisions that prioritized building on resources and relationships and respect for community assets and values.

## The Proposed Multisystemic Intervention Under Evaluation

**Fig. 1:**
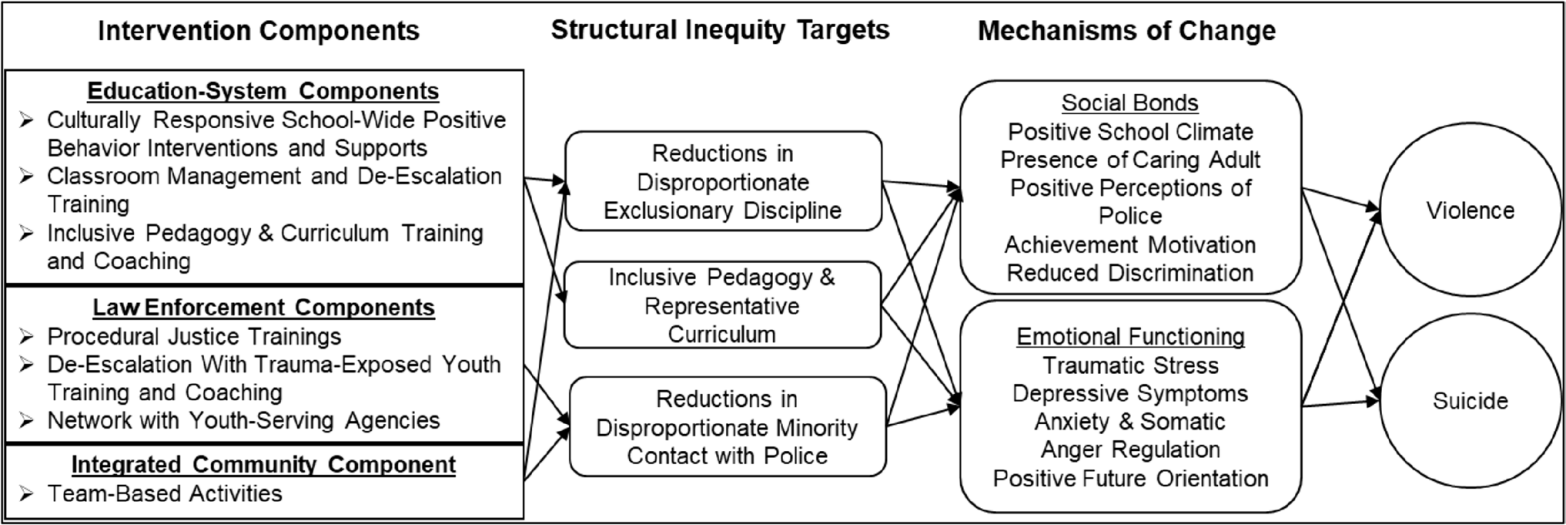
Underlying logic model to explain the process through which intervention components may address structural inequities, which in turn may reduce health disparities

Education and law enforcement systems are partners in the proposed intervention. Both systems are highly motivated to promote youth safety and have the positionality to actively move toward promoting equity. In addition, Black youth are often exposed to inequities within those systems. About 17% of Black American youth have been suspended from school, compared to about 5% of their White American counterparts (Losen & Gillespie, 2012). This difference is not explained by differences in their actual level of disruptive behavior (Skiba et al., 2002). Exclusionary discipline practices significantly increase the risk of incarceration, creating a school-to-prison pipeline in which youth feel targeted by and disconnected from school, lose instructional time, drop out, and become involved in the justice system (e.g., Novak, 2018; Skiba et al., 2002). Black youth are also exposed to cultural biases occurring in school curricula that promote Eurocentric culture, history, and values as “right,” while marginalizing the contributions of other cultures (e.g., Thompson & Neville, 1999). The combined effects of disproportionate exclusionary discipline and cultural bias in curriculum and pedagogy may cause some Black American youth to believe that school is neither relevant nor a viable path to success (Buhin & Vera, 2009). With respect to law enforcement, Black American youth are twice as likely to experience contact with the police compared to White American youth, and they experience disproportionately severe outcomes of that contact (Crutchfield et al., 2009). Black American youth are more likely to be arrested, referred to court, and placed in detention; they comprise 58% of youth who are sentenced to adult state prisons (e.g., Hockenberry & Puzzanchera, 2017). The well-established inequities of disproportionate exclusionary discipline, bias in curriculum and pedagogy, and disproportionate minority contact are concrete, operationalizable, and measurable factors that serve as the key targets of the proposed intervention (see Fig. 1).

The multisystemic, community-level intervention that is the focus of this project (Strengthening Opportunities for Achievement and Resilience; SOAR) consists of school, law enforcement, and integrated community interventions developed for implementation in a medium-sized city in the southern USA. The school-based intervention components include a culturally responsive, community-inclusive adaptation of School-Wide Positive Behavior Interventions and Supports; trauma-informed classroom management and de-escalation training for all school personnel; and universally engaging pedagogy and curriculum for all teachers. The law enforcement-based intervention components focus on improved coordination and systems-level integration among local youth-serving agencies and procedural justice interventions. Procedural justice interventions include training in disproportionate minority contact, and training and coaching on de-escalation with youth for police officers. All school personnel and police officers receive the initial trainings, and follow-up coaching is optional. The community intervention includes building pathways between police officers and youth-serving agencies to increase services and connectedness for high-risk youth, as well as team-oriented community-building between police officers, school personnel, and youth.

### Partnership as a Critical Foundation for Addressing Structural Inequities

Regardless of its potential to produce change, an intervention must be feasible, acceptable, and sustainable to have a meaningful and lasting impact (e.g., Glasgow et al., 1999). Partnership across systems is a key but underdiscussed foundation for effective prevention (Baum et al., 2006; Wallerstein & Duran, 2010). Specifically, representatives of partnering agencies should be involved in developing, implementing, and designing the evaluation of systems-level interventions.^1^ We took a transdisciplinary participatory action research approach for the development of this intervention (goal specification; logic model identification, and intervention development and testing; Mehari et al., 2023). Our intervention development took place within the context of long-standing partnerships and built on existing collaborative work. The investigators on this project and the school and police partners had already established a history of positive relationships and collaboration, largely due to the efforts of a police leader, who headed a citywide initiative to use a trauma-informed approach to reduce violence. We created goals at the intersection of community needs and priorities (which included youth violence, with an increasing concern about suicidality); existing partnerships; and opportunities and resources. During this time, the National Institutes of Health released a funding opportunity announcement focused on structural racism/discrimination in health disparities, providing an opportunity to address shared priorities, which we brought to our police partners’ and school partners’ attention.

The considerations that guided decision-making in the methods we describe included the need to respect partners’ core values, priorities, resources, and sociopolitical and economic contexts; the need to avoid stressing already overburdened systems; the limitations of the investigators’ expertise and resources; and the need to identify approaches that had an adequate evidence base.^2^ For example, the core of the law enforcement-based component was based on a training that the police captain of a precinct requested that our team develop to address a knowledge gap he identified related to working with adolescents among his officers (Mehari et al., 2023). During brainstorming to build out a larger strategy to reduce youth violence and suicidality, we engaged in a series of conversations with police leadership and officers. Across the board, one of the most frustrating situations officers identified related to juveniles was feeling “stranded” when they were in situations that required some type of intervention (i.e., they could not leave the juvenile alone), but that did not require an arrest or detention of the juvenile. Officers indicated that they often were in situations where no agency would take ownership (e.g., Child Protective Services, the juvenile court, the school system), and that these types of situations led to burnout. Based on these conversations, we concluded that increased partnership and networking across youth-serving agencies and creating space to facilitate conversations about delineation of responsibility across different situations would be a key component of the intervention. This approach would result in high-risk youth being quickly identified and referred for services. This is just one example of how partnership shaped the design of the intervention.

Logistically, the partnership involved monthly meetings between the investigator team and central office school partners, and between the investigator team and police partners (conflicting schedules made it impossible to meet all together every month). In turn, the school partners and police partners communicated directly with the captains and principals as well as vertically with upper leadership, especially prior to relationships being established between the investigators and other personnel in the schools and police department. When one agency had concerns about a particular approach, this was shared with the other agency, who collaboratively brainstormed. Each agency had the final decision about what would occur inside their agency. In our case, the specific agency representatives had trusting relationships with each other and respected each other’s input, so there were no major difficulties, but we recognize that there are difficulties in shared decision-making when relationships are more fraught. We also engaged in fun activities to build rapport. For example, after the police department indicated that the investigators should all go on ride-alongs in each of the precincts to ensure the relevance of the de-escalation trainings, one of the investigators hosted a *Ride Along* movie watch party at his house for all the school and police partners, so that the major in charge of field operations could provide commentary about appropriate and inappropriate ride-along behavior.

In addition to guiding major decisions like intervention content and measurement strategies, partnership considerations affected all other choices, including language usage. For example, the word “racism” is polarizing. The word, in its colloquial use, is a slur that locates the source of the problem in an individual, rather than describing a shared problem embedded in systems that is harmful to the well-being of the country as a whole. The use of the word tends to be adversarial as opposed to collaborative. In developing interventions, it is important to use a unifying shared language and conceptualization. One way to do this is to operationally define the problem or target in a manner that is acceptable across participating groups. Because of this, we used alternative language to the word racism; for example, our goals were framed as promoting proportionality in discipline, increasing universal engagement in pedagogy, strengthening procedural justice, and building positive relationships to support youths’ success.

## Design of the Evaluation

### Selecting and Defining Community Units

The community-level focus of the intervention necessitates that the community be the unit of analysis, which calls for careful consideration of how to define and select communities. Defining community-level units involves consideration of the size, characteristics of the residents, defined boundaries (e.g., natural boundaries, community identity), and scope and focus of intervention activities (Farrell et al., 2016). Communities need to be large enough to adequately capture the root causes and health outcomes targeted by the intervention, but not so large that they exceed the resources needed to provide adequate dosage. In terms of the design, communities need to be selected that are similar on key characteristics (e.g., size, socioeconomic status, sociopolitical context) and sufficiently autonomous to minimize potential diffusion of intervention activities. Another key challenge for this project was that a community unit needed to be defined in ways that made sense for both law enforcement and schools.

These factors ultimately led us to define community units based on the boundaries of police precincts, in which a school is co-located. The precincts are divided by highways and major roads, such that neighborhoods are intact within precincts. Defining the boundaries of community units by precincts within a city allows for the units to be adequately similar on key characteristics, small enough so that intervention resources are not overstretched, and sufficiently autonomous to reduce the likelihood of diffusion of intervention effects. In this city, there were four traditional precincts (excluding a downtown business precinct with little residential living); each represented a quadrant of the city and shared a border with two other precincts. Each precinct is commanded by a captain, who has some choice around selecting officers in their precinct and who reports to the major of field operations. Command staff make decisions about moving officers to different precincts based on staffing needs. We selected one middle school within each precinct’s boundaries to create four communities that each consisted of a police-precinct pair. Four schools were selected for the proposed intervention, including two predominantly Black American schools and two more ethnically diverse schools. In two precincts, there was only one public middle school in each, both over 85% Black American and serving low-income communities (indicated by 95% of students eligible for federally subsidized free lunches). There were two public middle schools in each of the remaining precincts, and we selected the most ethnically diverse schools (both between 45 and 55% White, about 30% Black, and between 10 and 12% Latine). Both had greater income diversity than the predominantly Black American schools (~ 55% to 80% eligible for free lunches). The variation in ethnic and income status composition has some tradeoffs. On the one hand, differences in composition add to other possible pre-existing differences between communities that may increase confounding factors in the evaluation of the intervention. On the other hand, having diversity across ethnic composition makes it possible to begin exploring the effectiveness of the intervention for Black American youth within predominantly Black American schools, and to assess reductions in disproportionality across White and Black American youth within ethnically diverse schools.

Including predominantly Black American communities is vital for any research on structural inequities, because indicators of structural inequities vary not only as a function of an individual’s ethnicity but also as a function of the ethnic composition of the larger community. The impact of centuries of injustice is most pronounced in highly segregated areas. For example, predominantly Black American schools have the highest rates of exclusionary discipline, and attending a predominantly Black American school is a major risk factor for suspension for Black American youth (e.g., McIntosh et al., 2021; Skiba et al., 2002). Determining how to assess change in disproportionality in fairly homogeneous environments poses a challenge. It is not possible to compare rates of exclusionary discipline between Black and White students within segregated schools (nearly 100% Black), although it will be possible to show overall reductions in exclusionary discipline. One potential strategy to assess proportionality is to compare findings against publicly available district-level and national-level averages disaggregated by race, gender, and race × gender, which may serve as a useful benchmark to identify how close the intervention comes to closing the gap in exclusionary discipline (e.g., McIntosh et al., 2018).

### Application of a Multiple Baseline Design to Assess Community-Level Impact

We selected a multiple baseline experimental design to assess the impact of the intervention on change at the community level. Multiple baseline designs are a particular type of single-case design in which individual units transition from a baseline phase to an intervention phase on a staggered schedule, and intervention effects are evaluated based on multiple observations collected during each phase (Kazdin, 1982). Multiple baseline designs have a long history of use for evaluating interventions in studies that focus on individuals as the unit of analysis (Slocum et al., 2022). Biglan and colleagues (2000) discussed the advantages of multiple baseline designs in which community-level units (e.g., neighborhoods), rather than individual people, are the unit of analysis. Multiple baseline designs in which community-level intervention effects are evaluated using data collected from multiple individuals within each cluster may be viewed as a type of stepped wedge cluster randomized trial (e.g., Rhoda et al., 2011). These two types of designs do, however, differ in their emphasis on several key aspects. A major requirement of multiple baseline designs is the collection of multiple waves of outcome data within each phase. This is not required by stepped wedge designs, which often include a single wave per phase. Whereas multiple baseline designs often assign a single cluster to each sequence, stepped wedge designs typically assign multiple clusters to each sequence. Finally, unlike stepped wedge designs that require obtaining outcomes for multiple individuals within each cluster, multiple baseline designs can examine outcomes assessed at the community (cluster) level, such as archival data (Masho et al., 2016). These differences make the label “multiple baseline experimental design” the most accurate way to describe this study’s design.

Our design involves collecting baseline data starting in the first year of the project. Implementation of the intervention is staggered such that it is initiated in one of the four communities at the start of year 2, in a second community in year 3, in a third community in year 4, and in the fourth community in year 5 (see Table 1). Once initiated, data collection and intervention activities continue in each community through the end of the project. We designed our project to meet the standards of evidence criteria for single-case, multiple baseline designs established by the Institute of Education Sciences based on a panel of experts in single-case intervention research designs (Kratochwill et al., 2013). Assigning the four communities to two phases (i.e., control and intervention) provides a total of eight phases. Although only one wave of data is collected from the fourth community during the intervention phase, six phases remain when this community is excluded, which meets the standards’ minimum requirement. Our design also exceeds the minimum requirement of six data points per phase for archival data for all communities, and for survey data for all but one of the communities (the first community to receive the intervention will only have three baseline data points for the survey). We followed several other recommendations included in the standards of evidence to increase the rigor of the design*.* These included randomly assigning the four communities to the sequence of implementation and evaluating outcomes based on statistical, rather than solely on visual, analysis. Consistent with the value of transparency, involving the partnering communities in the randomization process is important, and can also be playful and fun. We designed a kick-off celebration with school and precinct leadership during which a representative of each community chose a helium hot air balloon, with a sealed envelope attached to the balloon indicating the start date of the proposed intervention. In essence, this approach provides repeated experiments of a single-case design, providing corroborative evidence of effectiveness (Rapkin & Trickett, 2005).

**Table 1:** Overview of the multiple baseline design

Year 1	Year 2	Year 3	Year 4	Year 5
Communities randomized to intervention start year	Intervention initiated in community A	Intervention maintained in community A	Intervention maintained in communities A and B	Intervention maintained in communities A, B, and C
Planning and preparation to roll out the intervention		Intervention initiated in community B	Intervention initiated in community C	Intervention initiated in community D
Baseline survey data collected in all communities; archival data retrieved	Data collection continues in all communities	Data collection continues in all communities	Data collection continues in all communities	Data collection is completed in all communities

The focus of the intervention is change in community-level youth outcomes (i.e., 11- to 15-year-old youth in the middle schools, and youth 18 and under in the precincts). Outcomes will be assessed by both survey and archival data. We will administer measures to samples of students, school personnel, and police officers every 3 months (during each school year for school-based surveys; throughout the year for precinct-based surveys) from year 1 through the start of year 5. This will provide 13 waves of surveys from students and school personnel, and 17 waves from police officers. We will also obtain archival data from schools (e.g., disciplinary office referrals, suspensions, and expulsions) and precincts (e.g., dispatch calls, critical incidents) for the 3 years preceding the project and on a quarterly basis throughout the project. This will provide quarterly data for 22 waves of school-level and 29 waves of precinct-level archival data.

### Selection of Constructs and Measures

Several domains of constructs are needed to evaluate both the impact and the logic model of the intervention (see Fig. 1). The primary outcomes are actual occurrences of violence and suicidality. The secondary outcomes are the disproportionality of structural inequities targeted by the interventions (e.g., discipline practices; use of force). The other domains of constructs—integrity of implementation, including quality and fidelity; and hypothesized mechanisms of change—are included to address *how* the intervention was effective, if it was, and *why* it was not effective, if it was not. The primary and secondary outcomes and hypothesized mechanisms of change will be assessed during the baseline and intervention conditions. Integrity of implementation will only be assessed during the intervention condition.

To assess community-level health outcomes of youth violence and suicidality, we will collect (a) violent incidents from school discipline reports and police case reports; (b) youth report of school safety, experiences of peer victimization, exposure to community violence, and perpetration of aggression; and (c) teachers’ ratings of youth victimization and perpetration. Youth and teacher reports of youth victimization and perpetration will be used to estimate a latent variable of youth violence at the community level. Suicidality outcomes will be assessed by aggregate suicide protocols and suicide-related events from school counselor and administration records. Youths’ self-reports of suicide ideation and suicide attempts will also be aggregated at the community level. Youth self-report will be considered primary for suicidality. We will evaluate our hypotheses by examining the consistency of findings across measures, with stronger evidence for impact when findings are consistent across measurement.

The nature of structural inequities poses a particular challenge for measurement (see Table 2 for proposed options). For example, aggregate measures of self-reported prejudice or experiences of discrimination could simply point to interpersonal racism. Measuring prejudice or interpersonal racism by itself is not sufficient because the goal of this structural intervention is to reduce systemic disparities, rather than to change individuals’ attitudes or interactions outside of embedded power structures. We chose to focus on institutional inequities (operationally defined as disproportionality in exclusionary discipline based on race and disproportionate minority contact with police along the use of force continuum) and cultural responsiveness (nonrepresentativeness in curriculum). Youth contact with the police and school exclusionary discipline will be measured by archival. Representativeness in curriculum will be assessed by coding of lesson plans and classrooms by trained graduate students in education. Teachers will be randomly sampled for observations of lesson plans and classrooms. This approach allows for the integration of multiple sources, reducing the likelihood of biased information. It includes the collection of archival disciplinary data across schools, archival police records, and school-based observations. Making use of community-level archival data offers multiple advantages, including a long baseline against which to evaluate intervention effects (Masho et al., 2016).

**Table 2:** Measures to assess structural inequities

Measure	Source	Constructs
Exclusionary discipline practices	School archives	Rates of office discipline referrals, suspensions (in and out of school), and expulsions disaggregated by race and gender and by school
Disproportionate minority contact	Police archives	Rates of arrests, charges, and use of force towards youth (18 years old and younger) by precinct, disaggregated by race
Rubric for Culturally Responsive Lessons/Assignments (Aguilar-Valdez, 2015)	Observation	Use of responsive and inclusive teaching strategies and materials
Inventory of School Climate (Brand et al., 2003)	Youth self-report	Support for cultural pluralism in the school environment Perceived teacher support
Adolescent Discrimination Distress Index (Fisher et al., 2000)	Youth self-report	Perceived discrimination experiences based on race/ethnicity
Police Legitimacy Scale (Fine et al., 2020)	Youth self-report	Perception of police as trustworthy and just

To assess mechanisms of change (e.g., social bonds; emotional functioning), we will use a combination of archival data and aggregate survey responses from multiple sources including youth, school personnel, and police officers. The perspectives of youth will be assessed by surveys of students at the participating schools. In year 1, we will recruit 600 students randomly sampled from the 6th, 7th, and 8th grade rosters of each school (*N* = 150 per school; 50 students per grade). In each subsequent year, we will recruit students from the cohort of incoming 6th graders and from a random sample of 7th and 8th grade students to replace those lost to attrition (estimated 10%). This will enable us to maintain a sample of 150 students to represent each school during each of the 5 years of the project (total *N* ~ 1560). We will ask homeroom teachers to complete ratings of each participating student. To assess mechanisms of change, youth will report on their perceptions of school climate, their experiences of discrimination, and their relationships with adults, including teachers, school personnel such as school resource officers, and the police. They will also provide information about their emotional functioning (e.g., symptoms of traumatic stress, somatic complaints such as difficulty sleeping, perceived burdensomeness, positive outlook, achievement motivation).

Implementation integrity is vital to support the basic premise that we are evaluating the impact of the program; we have to establish that key components of the intervention are happening (adherence), with adequate dosage and quality of delivery, and reasonable engagement of participants (cf., Bumbarger, 2014). We will evaluate integrity of delivery through several indicators, including observations (such as the Schoolwide Evaluation Tool; Horner et al., 2009), occurrence of activities (trainings, coaching sessions, events), the proportion of eligible staff who participate by activity (dosage), and participants’ reports of quality of delivery and their own satisfaction with the activities.

### Statistical Analysis

We hypothesized that implementation of the intervention in each community would be associated with (1) greater decreases (or slower increases) in youth violence and suicidality compared to their baseline trajectories, and (2) greater decreases in indicators of structural inequities compared to their baseline trajectories; and that (3) the observed changes would coincide with greater increases in community-level social bonds and positive emotional functioning compared to baseline trajectories. For intervention communities that were ethnically diverse, we hypothesized that changes in youth violence and suicidality would be more pronounced among Black youth compared to White youth in the community.

We will use multilevel models to examine community-level intervention effects on each outcome. Ferron (1997) recommended the use of multilevel models for analysis of multiple baseline data because they (a) are flexible enough to handle dependent error structures and treatment effects that vary over time and community units; (b) allow the use of data from multiple community-level units in a single analysis; and (c) can accommodate outcomes that are not normally distributed (e.g., binomial models for count data). Monte Carlo simulations applying these models to multiple baseline data have suggested that accurate confidence intervals can be obtained for estimates of treatment effects as long as the serial dependency in the errors is modeled and the Kenward-Roger approach is used to estimate degrees of freedom (e.g., Ferron et al., 2009).

1 $$\mathrm{Level \,1}\!: {Y}_{ti}={\beta }_{0i}+{\beta }_{1i}{Year}_{ti}+{\beta }_{2i}{Season}_{ti}+{\beta }_{3i}{Int1}_{ti}+{\beta }_{4i}{Int2}_{ti}+ {e}_{ti}$$

2 $$\mathrm{Level \,2}\!: {\beta }_{0i}={\gamma }_{00}+{\gamma }_{01}{C1}_{i}+{\gamma }_{02}{C2}_{i}+{\gamma }_{03}{C3}_{i}+ {U}_{0i}$$

$${\beta }_{1i}={\gamma }_{10};\, {\beta }_{2i}={\gamma }_{20}; \,{\beta }_{3i}={\gamma }_{30}; \,{\beta }_{4i}={\gamma }_{40}$$ where $${e}_{ti} \sim N(0,\,{\sigma }^{2},\,\mathrm{ and }\,{U}_{0i}\sim N(0,\,{\tau }_{00})$$  

Our analysis plan for the survey data collected from students, school personnel, and police will be similar to the analyses for a project that used a multiple baseline design to evaluate the impact of a community-level violence prevention program in three urban communities (Farrell et al., 2018). Our level 1 within-person model (see Eq. 1) will represent observations of outcomes, ${Y}_{ti}$, at time $t$ for individual $i$ as a function of parameters that attempt to capture systematic changes across the multiple waves of data and the influence of intervention effects. Models will specify an autoregressive covariance structure. Our initial analysis will model change across waves as a function of a random intercept that varies across persons, fixed effects for linear change both across project years (*year* = 0 to 4) and within each year (*season* =  − 0.50, − 0.25, and 0 for fall, winter, and spring, respectively), and intervention phase; and a time-specific and person-specific residual. We will evaluate models for each outcome based on how well they fit the data, including comparisons to more parsimonious models (e.g., simple linear trend across all waves; intercept-only models). Intervention phase will be represented by two dummy-coded variables using baseline phase as a reference (*Int1* = 1 if year 1 of implementation; *Int2* = 1 if years 2 to 4 of implementation).

The level 2 model (see Eq. 2) expresses between-person differences in intercepts as a function of an overall intercept, the community where the individual lives, and a residual (${U}_{0i}$), representing between-person differences in intercepts not accounted for by the community. Community is dummy-coded with community 4 (randomized to receive the intervention last) as the reference (C1 = 1 if community 1; C2 = 1 if community 2; C3 = 1 if community 3). The level 2 model also includes fixed effects for year (${\gamma }_{10})$, season (${\gamma }_{20})$. The coefficient ${\gamma }_{30}$ represents intervention effects on outcomes during the first year of implementation, ${\gamma }_{40}$ represents effects in subsequent years of implementation, and ${\gamma }_{40}$ – ${\gamma }_{30}$ can be estimated to evaluate differences in effects between the first and subsequent years of implementation. This will enable us to compare changes during each intervention phase within each community relative to its baseline trajectory, and to compare communities receiving the intervention with changes in communities not receiving the intervention during that same period (i.e., the counterfactual).

Given the small number of communities, we will use the Kenward-Roger approximation for degrees of freedom (e.g., Ferron et al., 2009). We will estimate effect sizes for the intervention (Cohen’s *d*). As in our previous project (Farrell et al., 2018), we can also explore incorporating additional effects into the model. For example, participants’ sex and grade could be included in the level 2 model to explore potential sex and grade differences in intervention effects on student outcomes. To supplement our statistical analysis, we will also use visual analysis of multiple baseline design graphs following criteria recommended by Kratochwill et al. (2013). This will provide a more conservative indicator of change in multiple baseline designs and increase face validity (Wolfe et al., 2019).

A key consideration in the design of this project was determining the sample size needed to ensure adequate statistical power to detect community-level effects. We addressed this by conducting a power analysis using swdpwr, an interactive web application developed in R (Chen et al., 2022). Although developed for stepped wedge designs, swdpwr is more generally applicable to generalized linear mixed models that include a treatment effect and a fixed time effect, making it appropriate to apply to this multiple baseline design. The treatment effect within swdpwr is represented by a main effect during an intervention phase relative to a baseline phase. Because our analytic model includes separate parameters to represent effects that occurred during the first year of implementing the intervention (${\beta }_{3i}$), and intervention effects during subsequent years (${\beta }_{4i}$), we ran separate sets of models to estimate power for each of these effects (see Supplemental Materials for a detailed description). We estimated power using a range of values for within-period correlations (0.01, 0.10), between-period correlations (0.005, 0.05), and within-individual correlations (0.10 to 0.50). We evaluated power to detect a range of effect sizes including those between small and medium effects (*d* = 0.30 and 0.40), medium effects (*d* = 0.50), and large effects (*d* = 0.80) based on Cohen’s criteria (1977). For models assuming a within-period correlation of 0.01, power was consistently high for effect sizes as low as 0.30 for student surveys (power = 0.1.00), school personnel surveys (power = 0.80 to 0.95), and police surveys (power = 1.00). For models assuming a within-period correlation of 0.10, adequate power was obtained for effects as low as 0.40. These power estimates are consistent with the findings of a previous study that used a similar design with three middle schools (Farrell et al., 2018). Analyses of three waves of teacher ratings of students per year across 5 years identified significant intervention effects as small as *d* = 0.21.

## Strengths and Weaknesses of a Multiple Baseline Design Relative to Alternative Designs

There are important strengths and limitations of our proposed multiple baseline design compared with alternative designs. By comparing changes following implementation of the intervention to the pre-intervention baseline trajectory, we can evaluate the community-level impact of the intervention by allowing each community to serve as its own control. This makes it possible to examine the patterns of change over time, the cumulative impact of the intervention across multiple years of implementation, and variability in rates of change across different outcomes. It also makes it possible to test whether other events in time (e.g., a pandemic; change in policy or law) better explain patterns over time than the onset of the intervention. Our design and analytic plan also allow us to compare intervention effects during the first year of the intervention and the extent to which they are sustained or increase during subsequent years of implementation. This is particularly important as some community-level intervention effects may not emerge until two or more years of implementation (Farrell et al., 2018; Sullivan et al., 2021). Our design also provides multiple replications, which makes it possible to determine the consistency of intervention effects across communities that implement the intervention at different points in time. This also mitigates history-related threats to validity.

Multiple baseline designs also have practical and values-based advantages. From a practical standpoint, staggering the start of an intervention across multiple project years makes it possible to focus on introducing the intervention in one community at a time, incorporate lessons learned into a more streamlined intervention as we move into each additional community (i.e., systematic replication), and tailor the implementation to contextual factors that vary across communities (Biglan et al., 2000). Another strength is the randomization to start time, rather than selecting the most prepared communities to start first, which introduces readiness bias. Randomization also essentially creates multiple small trials. This creates an opportunity to combine the results of these studies with other small trials, in a process described as cumulative trials, to essentially create a large randomized controlled trial over time (e.g., Wyman et al., 2015). From a values-based and partnership perspective, multiple baseline designs have an advantage in that every participating community will eventually receive the intervention and its associated resources. Moreover, once initiated, intervention activities continue through the end of the project. This provides a stronger basis for sustainability and is highly acceptable to partnering communities because it is consistent with social justice and equity values.

In contrast to multiple baseline designs, randomized clinical trials (RCTs) are often considered a “gold standard” for determining intervention effects. They do, however, have significant limitations when used to evaluate community-level interventions. A cluster randomized design, in which community units rather than individuals are randomized to experimental conditions, would require a large number of communities to have adequate power to detect an intervention effect. Hemming and colleagues (2011) estimated that a design that assigned 15 communities per condition (i.e., 30 total) would have a power of only 0.62 to detect a 10% difference in outcomes. Our approach allows us to conduct a rigorous test with fewer communities, which enables us to concentrate our intervention resources to maximize the likely impact. In terms of acceptability, half of the participating communities in an RCT design would not receive the intervention, which actively counteracts the project goal of promoting equity.

Although a multiple baseline design addresses many threats to internal and external validity, it has significant limitations, including threats related to history or diffusion. The focus on a small number of communities means that any significant change (e.g., new principal, community-wide racialized event) could affect the results. Such changes (history effects) could result in reductions in the impact of the intervention (e.g., a new principal whose commitment to the intervention components is low) or increases in the impact of the intervention (e.g., community-wide outreach and engagement). Any possible history effects that occur within such an intervention should be monitored, carefully documented, and reported when disseminating findings. Although our design does not allow us to eliminate such effects, it provides us with a basis for testing for such effects by determining if intervention effects are replicated across communities. Another weakness is that intervention effects are confounded with time, with more communities receiving the intervention as time goes on. As such, if an event happens at the national or local level that slowly results in increases or decreases in violence or suicidality, it will be difficult to determine the impact of the intervention.

Another possibility is that the intervention will be disseminated to communities outside of the randomized timing of intervention initiation (diffusion effects). For example, schools that have been asked to wait three to four years for the intervention may choose to implement similar (or different) strategies to address the targets of the intervention, rather than wait for their randomly assigned start year. At a smaller level, teachers and police officers might transfer from a school/precinct in the intervention phase to one in the control phase, or share information with colleagues in communities not yet receiving the intervention who then try to make use of the strategies. If that is the case, other communities could begin to see impacts of the intervention without officially receiving the intervention. Although we recognize that the majority of schools implement some kind of violence prevention program, they are often implemented with low quality (Finkelhor et al., 2014). The presence of such programs does not, however, detract from the goal of this project, which is to assess whether this intensive, community-level experimental intervention performs significantly better than what sites will do on their own (their pre-intervention baseline). In our case, the partnership team will work closely with site leaders to develop a shared understanding of the issue. We will fully acknowledge the real problem of children dying and express our hope to the site leaders that with the support that external funding and the team’s full effort can provide, the intervention may be worth the wait. So far, site leaders have been willing to align their strategic plans with the randomization of SOAR so that SOAR is part of their own planned roll-outs for their site. As with history effects, any and all related interventions or programming that the sites implement before and during the intervention periods will be monitored, carefully documented, and reported when disseminating findings.

Given the need to select from a small number of possible communities, it is possible that baseline differences across communities will lead to differential impacts of the intervention. In some respects, baseline differences in a multiple baseline design are less of a concern than in a pure between-cluster design because the analyses focus on both within-community and between-community differences in trajectories. The small number of participating community units has limitations related to estimating the community-level intercepts with random effects, and it may only be possible to estimate the intercepts with fixed effects. We will examine the consistency of intervention effects within each community, which will enable us to explore the extent to which baseline differences influence outcomes. However, the small number of communities will limit our ability to use statistical analyses to evaluate the consistency of intervention effects across communities or determine which community-level factors moderated intervention impact. In addition, we used the most proximal established power analytic approach for our design (swdpwer), which was created for stepped wedge design. This approach may not fully take into account the real-world complications of a multiple baseline design and community-level interventions, so the power analysis may not be entirely accurate.

Additionally, because the proposed intervention includes multiple components, it will not be possible to determine each components’ contribution to changes in outcomes. We considered designs in which components of the intervention, rather than the whole package, were randomly assigned to communities. This includes designs such as multiphase optimization strategies (MOST) and cluster-based cross-over designs (Arnup et al., 2017; Collins et al., 2005). MOST and similar designs have multiple advantages, including flexible and adaptive designs that identify the key active components of interventions and the optimal dosage for maximum impact. Although MOST designs can be integrated into multiple baseline designs, we decided not to do so for several reasons. Given previous research on community-level interventions for youth violence, we anticipated that those interventions may need to be in place for several years prior to produce detectable changes at the systems level (e.g., Farrell et al., 2018). In order to then fully test and compare different combinations of intervention components, we would have needed to intervene in a much larger number of schools, which we were not sufficiently resourced to do. Should this overall multisystemic approach prove to be effective, future research may focus on teasing out the contribution of specific components. Toward that end, we included measures to assess changes on specific constructs targeted by each component (see Fig. 1 and Table 2). This will provide an indication of the extent to which each intervention component is producing its intended effect on the specific targets of change.

## Recommendations for Improving Health Disparities Research

1. Build a sense of urgent, shared public need to address health disparities. There seems to be a shared belief that only members of marginalized groups are harmed by health disparities, and that these health disparities are perpetrated by White men. This is despite evidence that our whole country is harmed by structural inequities (e.g., Kennedy et al., 1996). We need to come to a shared language that is more universally acceptable and does not localize the problem within one group. Systems-level work is always difficult due to all the moving pieces within systems, and work on health disparities can make people feel vulnerable and uncomfortable. Changing the narrative to build a sense of shared purpose and shared effort may make more systems willing to partner, despite the hard work.
2. Respect the validity of values- and partnership-based decision-making. There is an inherent tension between the desire for strong internal validity and the need for respectful partnerships and values-congruent work. For example, the value of shared decision-making may often be in conflict with researchers’ efforts to maintain scientific rigor by controlling all aspects of the design and evaluation. To a smaller degree, there is a tension between the need to determine efficacy (dependent on internal validity) and the importance of the acceptability, feasibility, and scalability of both the intervention approach and the evaluation approach. The depth and range of health disparities in the USA can only be addressed when individuals in multiple systems commit to enacting coordinated change within systems. As such, values-based decisions and true partnerships are equally important to scientific rigor.
3. Promote community-based and systems-level partnerships in grant funding to promote the quality and quantity of health disparity research. For example, funding that emphasizes systems-level change or cross-systems partnerships could be released. Scientists applying for funding should be encouraged to include leaders in other systems as co-investigators. Criteria for evaluating investigators’ qualifications to do the work could then be extended to contributions to policy, practice, and the community, rather than just contributions to science. It is hoped that such efforts would push the research out of scientific siloes and move into systems-level adoption and sustainable change.

### Supplementary Information

Below is the link to the electronic supplementary material.

Supplementary file1 (DOCX 28 KB)

## Data Availability

No new data were created or analysed in this study. Data sharing is not applicable to this article.

## Footnotes

1 These ideas were not generated by the authors, but represent wisdom and correction we have received from community leaders and from other, more experienced researchers who partner with communities.

2 Ibid.
