# Supplementary material for: Challenges in Evaluating a Community-Level Intervention to Address Root Causes of Youth Violence
Source: Prev Sci. 2024 May 11;25(5):774–85. doi: 10.1007/s11121-024-01678-7 (PMC11321925; doi:10.1007/s11121-024-01678-7)
Supplement: Supplementary file 1 — Supplementary file1 (DOCX 28 KB) [file 11121_2024_1678_MOESM1_ESM.docx]

**Supplemental Materials: Power Analysis**

We conducted a power analysis using the online version of swdpwr (<https://jiachenchen322.shinyapps.io/swdpwr_shinyapp/>), an interactive web application developed in R (Chen et al., 2022). Although designed for conducting power analyses for stepped wedge designs, swdpwr is applicable for generalized linear mixed models that include a treatment effect and a fixed time effect. It can accommodate continuous variables and cohort designs. It evaluates power based on the design of the study, size of the treatment effect, and values for random effects using a two-sided Wald-type test in which the variance of the treatment effect is based on maximum likelihood estimation. This approach was developed to address limitations of previously developed approaches for estimating power for stepped wedge designs (see Chen et al., 2022 for details). The treatment effect within swdpwr is represented by a main effect during an intervention phase relative to a baseline phase. Within our model, there are separate treatment effects represented by $\beta_{3i}$, which represents intervention effects on outcomes during the first year of implementation, and $\beta_{4i}$, which represents intervention effects during subsequent years of implementation. We ran separate sets of models to estimate power for each of these effects.

We conducted power analyses for our project based on its design, which was based on 4 clusters (i.e., schools or precincts). We specified a Type I error rate of *p* < .05, a cohort design, Gaussian family for responses, an identity link function for continuous measures, a conditional model (i.e., mixed effects model), and a marginal variance of 1.0 for outcomes. Although we specified values for the starting and ending mean response rates for the control group (1.20 and 1.25, respectively), Chen et al. (2022) noted that power calculations for continuous outcomes do not depend upon the parameters specified for the intercept or linear change. We estimated power based on 150 students per cluster and 13 waves of data for the student survey outcomes, 20 school personnel per cluster and 13 waves of data for the school personnel survey outcomes, and 50 police officers per cluster and 17 waves of data for police outcome measures.

We estimated power under a range of conditions. We specified within-period correlations of .01 and .10, between-period correlations based on half the value of the within-period correlations (this represents the default), and within-individual correlations ranging from .10 to .50. For each combination of within-period and within-individual correlations we estimated power to detect effect sizes between small and medium (i.e., *d* = .30 and .40), medium effects (*d* = .50), and large effect sizes (*d* = .80) based on Cohen’s criteria (1977).

For our analysis of student surveys, power estimates across the 10 combinations of variance estimates ranged from .71 to 1.00. All were above .90 except for models assuming an effect size of .30 and a within-period correlation of .10. Power estimates for Year 1 intervention effects for the analysis of school personnel surveys were above .80, except for models assuming an effect size of .30 and a within-period correlation of .10. For analyses of intervention effects in subsequent years, all power estimates exceeded .80 except for models assuming effect sizes of .40 or less and a within-period correlation of .10. Power estimates for the analysis of police reports ranged from .73 to 1.00. All were above .90 except for models assuming an effect size of .30 and a within-period correlation of .10. In summary, our estimates suggest we would have sufficient power to detect medium effect sizes of .50 or larger for each source of outcome measure across all the combinations of correlations we considered, and small-to-medium effects under most of combinations of correlations we considered.

| Correlations | | | Effect size for implementation year 1 | | | | Effect size for implementation year 2 | | | |
| --- | --- | --- | --- | --- | --- | --- | --- | --- | --- | --- |
| Within-  period | Between-  period | Within-  individual | .30 | .40 | .50 | .80 | .30 | .40 | .50 | .80 |
| Student Survey Data (cluster size = 150) | | | | | | | | | | |
| .01 | .005 | .10 | 1.00 | 1.00 | 1.00 | 1.00 | 1.00 | 1.00 | 1.00 | 1.00 |
| .01 | .005 | .20 | 1.00 | 1.00 | 1.00 | 1.00 | 1.00 | 1.00 | 1.00 | 1.00 |
| .01 | .005 | .30 | 1.00 | 1.00 | 1.00 | 1.00 | 1.00 | 1.00 | 1.00 | 1.00 |
| .01 | .005 | .40 | 1.00 | 1.00 | 1.00 | 1.00 | 1.00 | 1.00 | 1.00 | 1.00 |
| .01 | .005 | .50 | 1.00 | 1.00 | 1.00 | 1.00 | 1.00 | 1.00 | 1.00 | 1.00 |
| .10 | .050 | .10 | .76 | .94 | 1.00 | 1.00 | 0.71 | 0.92 | 0.99 | 1.00 |
| .10 | .050 | .20 | .76 | .95 | 1.00 | 1.00 | 0.71 | 0.92 | 0.99 | 1.00 |
| .10 | .050 | .30 | .77 | .95 | 1.00 | 1.00 | 0.72 | 0.92 | 0.99 | 1.00 |
| .10 | .050 | .40 | .77 | .95 | 1.00 | 1.00 | 0.72 | 0.92 | 0.99 | 1.00 |
| .10 | .050 | .50 | .78 | .95 | 1.00 | 1.00 | 0.73 | 0.93 | 0.99 | 1.00 |
| School Personnel Survey Data (cluster size = 20) | | | | | | | | | | |
| .01 | .005 | .10 | .87 | .98 | 1.00 | 1.00 | 0.80 | 0.96 | 1.00 | 1.00 |
| .01 | .005 | .20 | .88 | .99 | 1.00 | 1.00 | 0.82 | 0.97 | 1.00 | 1.00 |
| .01 | .005 | .30 | .90 | .99 | 1.00 | 1.00 | 0.85 | 0.98 | 1.00 | 1.00 |
| .01 | .005 | .40 | .92 | 1.00 | 1.00 | 1.00 | 0.89 | 0.99 | 1.00 | 1.00 |
| .01 | .005 | .50 | .95 | 1.00 | 1.00 | 1.00 | 0.93 | 1.00 | 1.00 | 1.00 |
| .10 | .050 | .10 | .56 | .80 | .94 | 1.00 | 0.50 | 0.75 | 0.91 | 1.00 |
| .10 | .050 | .20 | .57 | .82 | .95 | 1.00 | 0.52 | 0.77 | 0.92 | 1.00 |
| .10 | .050 | .30 | .59 | .83 | .96 | 1.00 | 0.54 | 0.79 | 0.93 | 1.00 |
| .10 | .050 | .40 | .62 | .85 | .96 | 1.00 | 0.57 | 0.81 | 0.94 | 1.00 |
| .10 | .050 | .50 | .64 | .87 | .97 | 1.00 | 0.59 | 0.83 | 0.95 | 1.00 |
| Police Data (cluster size = 50) | | | | | | | | | | |
| .01 | .005 | .10 | 1.00 | 1.00 | 1.00 | 1.00 | 1.00 | 1.00 | 1.00 | 1.00 |
| .01 | .005 | .20 | 1.00 | 1.00 | 1.00 | 1.00 | 1.00 | 1.00 | 1.00 | 1.00 |
| .01 | .005 | .30 | 1.00 | 1.00 | 1.00 | 1.00 | 1.00 | 1.00 | 1.00 | 1.00 |
| .01 | .005 | .40 | 1.00 | 1.00 | 1.00 | 1.00 | 1.00 | 1.00 | 1.00 | 1.00 |
| .01 | .005 | .50 | 1.00 | 1.00 | 1.00 | 1.00 | 1.00 | 1.00 | 1.00 | 1.00 |
| .10 | .050 | .10 | .79 | .96 | 1.00 | 1.00 | 0.73 | 0.93 | 0.99 | 1.00 |
| .10 | .050 | .20 | .80 | .96 | 1.00 | 1.00 | 0.75 | 0.94 | 0.99 | 1.00 |
| .10 | .050 | .30 | .81 | .97 | 1.00 | 1.00 | 0.76 | 0.94 | 0.99 | 1.00 |
| .10 | .050 | .40 | .82 | .97 | 1.00 | 1.00 | 0.77 | 0.95 | 0.99 | 1.00 |
| .10 | .050 | .50 | .83 | .97 | 1.00 | 1.00 | 0.78 | 0.96 | 1.00 | 1.00 |
